# Design of Shape Memory Thermoplastic Material Systems for FDM-Type Additive Manufacturing

**DOI:** 10.3390/ma14154254

**Published:** 2021-07-30

**Authors:** Paulina A. Quiñonez, Leticia Ugarte-Sanchez, Diego Bermudez, Paulina Chinolla, Rhyan Dueck, Truman J. Cavender-Word, David A. Roberson

**Affiliations:** 1 Polymer Extrusion Lab, The University of Texas at El Paso, El Paso, TX 79968, USA; paquinonez@miners.utep.edu (P.A.Q.); lugarte2@miners.utep.edu (L.U.-S.); dbermudez2@miners.utep.edu (D.B.); pchinolla@miners.utep.edu (P.C.); radueck@miners.utep.edu (R.D.); tjword@miners.utep.edu (T.J.C.-W.); 2 Department of Metallurgical, Materials and Biomedical Engineering, The University of Texas at El Paso, El Paso, TX 79968, USA

**Keywords:** material design, fused filament fabrication, shape memory polymers, melt compounding, scanning transmission electron microscopy, glass transition temperature, impact modifiers

## Abstract

The work presented here describes a paradigm for the design of materials for additive manufacturing platforms based on taking advantage of unique physical properties imparted upon the material by the fabrication process. We sought to further investigate past work with binary shape memory polymer blends, which indicated that phase texturization caused by the fused filament fabrication (FFF) process enhanced shape memory properties. In this work, two multi-constituent shape memory polymer systems were developed where the miscibility parameter was the guide in material selection. A comparison with injection molded specimens was also carried out to further investigate the ability of the FFF process to enable enhanced shape memory characteristics as compared to other manufacturing methods. It was found that blend combinations with more closely matching miscibility parameters were more apt at yielding reliable shape memory polymer systems. However, when miscibility parameters differed, a pathway towards the creation of shape memory polymer systems capable of maintaining more than one temporary shape at a time was potentially realized. Additional aspects related to impact modifying of rigid thermoplastics as well as thermomechanical processing on induced crystallinity are also explored. Overall, this work serves as another example in the advancement of additive manufacturing via materials development.

## 1. Introduction

Over the course of the past two to three decades, the technology of additive manufacturing (AM) has undergone a rapid metamorphosis from a budding novel prototyping tool to a blooming fabrication method. As interest in AM has grown, so too has the advancement of technologies based on this manufacturing technique where the feedstock type encompasses the main material categories of metals, polymers, and ceramics. The advancement of these technologies depends significantly on the availability of materials that possess a wide range of physical properties compatible with a given AM technology. The use of fused deposition modeling (FDM™) technology has increased due to its ease of use, its low cost, and the availability of feedstock materials, which are mainly common thermoplastics such as acrylonitrile butadiene styrene (ABS), polycarbonate (PC), polylactic acid (PLA), and other polyesters such as glycol-modified polyethylene terephthalate (PETG). Perhaps the greatest enabler for the proliferation of thermoplastic extrusion based AM platforms was the expiration of patents [1] on original FDM™ technology in 2009. Since then, virtually countless platforms based on FDM™ have emerged leading to other monikers, such as fused filament fabrication (FFF) and material extrusion additive manufacturing (MEAM), to be used when referring to the technology. As a result, AM platforms based on thermoplastic extrusion can now be found in academic, industrial, and home-user settings [2,3,4]. 

As societal expectation for FDM™-type AM platforms to be capable of meeting the demands of a wide range of applications grows, the need for the development of new materials that possess specific and sometimes boutique physical attributes has arisen. In general, there are three main strategies used for the development of new material systems for FDM™-type AM platforms: (1) compounding one or more constituents in the creation of novel polymer blends; (2) compounding filler materials with a specific desired physical property with a suitable polymer base, effectively creating novel polymer matrix composites [5]; and (3) synthesizing compatible materials. A fourth potential strategy was exemplified by Fenner Drives, who repurposed a thermoplastic urethane (TPU) originally developed for automated teller machine (ATM) drive belts. This material has been marketed as NinjaFlex^®^, a flexible FFF-compatible material widely used among home-use 3D printing hobbyists [6]. 

There is an ever-growing effort in academia in the development of application-specific materials systems for FDM-type platforms. One example was carried out by Masood and Song who developed metal-loaded composites that were compatible with FDM and intended for the creation of injection mold tooling [7]. A work by Khatari et al. demonstrated a composite based on ABS that was loaded with barium titanate (BaTiO_3_) and intended for dielectric applications [8]. There are also examples of the synthesis of new materials FDM™-type platforms that involved the development of urethane-based materials as has been demonstrated in efforts published by Harynska et al. [9] and Schimpf et al. [10]. Efforts in the creation of novel polymer blends for FDM™-type AM can also be found including works to augment ABS by combining it with other constituents such as styrene acrylonitrile (SAN) and styrene ethylene butylene styrene (SEBS) [11,12]. Another example of blends created using the thermoplastic rubber, SEBS, was demonstrated by Banerjee et al. who created a SEBS/polypropylene (PP) blend compatible with FDM™-type AM platforms. 

Work by the Polymer Extrusion Lab (PEL) at The University of Texas at El Paso (UTEP) has contributed to the widespread effort in academia in the creation of novel material systems capable of propelling the applicability of FDM™-type AM technology to new heights. We presented an overview elsewhere [13] and highlighted several efforts entailing the creation of polymer composites and polymer blends for FDM™-type AM. For example, Shemelya et al. [14] entailed the demonstration of a PC/tungsten composite system that was developed for space-based radiation shielding applications. Our lab has also completed several efforts in the development of FFF-compatible polymer blends. Early work entailed the development of binary blends composed of ABS and SEBS as well as ternary blends composed of ABS, SEBS, and ultra-high molecular weight polyethylene (UHMWPE) as first demonstrated by Rocha et al. [15]. Further refinement of the ABS:SEBS blend was demonstrated in Siqueiros et al. [16] where we began incorporating SEBS with a maleic anhydride graft (SEBS-g-MA) in an effort to better compatibilize the two thermoplastics. Several iterations of the ABS:SEBS-g-MA blend were created with the end result being a version capable of sustaining percent elongation values of 1506.6 ± 90.1%, which, to the best of our knowledge, is the highest sustained for a FFF/FDM™-fabricated material. Further experimentation with this blend system led to the finding that it possessed shape memory properties, which has led to the subject matter of the work presented here. 

### Shape Memory Polymers in FDM-Type Additive Manufacturing

There have been several efforts conducted by others in the development of shape memory polymer (SMP) materials for FFF additive manufacturing platforms. These efforts have dealt mainly with either integrating pre-existing polyurethane materials with FFF platforms or utilizing PLA-based materials. For example, two notable works involving DiAPLEX (SMP Technologies, Inc., Tokyo, Japan) were performed by Yang et al. [17] and Raasch et al. [18] where the shape memory effect of FFF-created objects was demonstrated. The development of PLA-based shape memory composite materials for biomedical applications was demonstrated by Pandey et al. [19] who assessed the shape memory properties of FFF-fabricated scaffolds composed of chitosan-filled PLA. Senatov et al. [20] performed a characterization effort of the shape memory properties of a PLA/hydroxyapatite composite, where the structure was also a FFF-created scaffold intended for biomedical applications. Multi-material FFF has also been demonstrated in literature by Estelle et al. [21] who demonstrated the shape memory properties of a structure that was essentially a square tubular structure with an outer shell of TPU filled with polycaprolactone (PCL).

While originally developed for AM processing, the ABS:SEBS blend presented in Siqueiros et al. was not designed by our group with intentions of using it as a shape memory material. However, it was demonstrated by Chávez et al. [22] that two iterations of the ABS:SEBS blend (50:50 and 25:75 by weight ratio ABS:SEBS) possessed shape memory characteristics meaning that a shape memory polymer that was compatible with FDM™-type AM platforms had been successfully developed. Key aspects of this previous work include: (1) finding that the polymer phases aligned due to the printing process; (2) the shape memory characteristics, namely shape fixation ratio (*R_f_*) and shape recovery ratio (*R_r_*) were dependent on raster pattern; and (3) that dependence of either *R_f_* and *R_r_* on raster pattern changed depending on deformation temperature. Additionally, it was reported that these shape memory properties were also dependent on SEBS content. In this and other works found in literature, *R_f_* and *R_r_* are calculated as follows [23,24,25]:

(1) $$R_{f}\left(\%\right)=\frac{\varepsilon_{u}}{\varepsilon_{m}}\times 100\%$$

(2) $$R_{r}\left(\%\right)=\frac{\varepsilon_{m}-\varepsilon_{p}}{\varepsilon_{m}}\times 100\%$$

where deformation is performed in a tensile testing machine and *ε_u_* is the elongation of the specimen after the load is removed, *ε_m_* is the maximum strain the specimen is subjected to (usually 100% elongation) and *ε_p_* is the elongation of the specimen after recovery. In most cases involving thermoplastic shape memory polymers, recovery is achieved by heating the specimens. Overall, shape memory effect can be assessed by the shape memory index (*SMI*), which is a combination of the two parameters calculated above and attained by the following equation [22,23]:

(3) $$SMI\left(\%\right)=(R_{r}\times R_{f})\times 100\%$$

**Figure 1:**
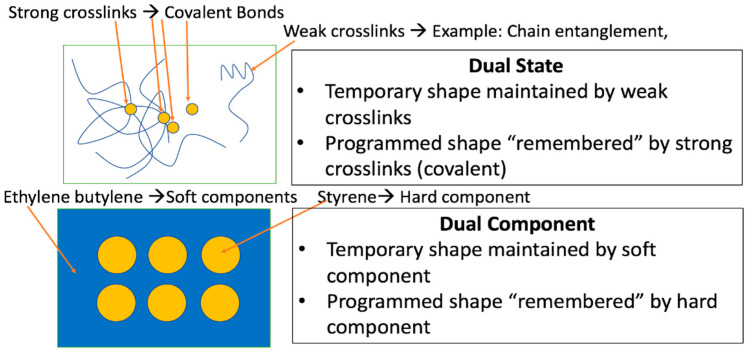
Depiction of the differences between dual state and dual component shape memory mechanisms. From [27].

Shape memory behavior in polymers has been classified into three distinct mechanisms by Yang et al. [26]: (1) the dual state mechanism; (2) the dual component mechanism; and (3) the partial transition mechanism. In the dual state mechanism, the shape memory effect is driven by strong crosslinks (covalent bonds) that control or “memorize” the permanent shape and weak crosslinks such as chain entanglement that hold the temporary shape. The dual component mechanism is characterized by physically hard and soft components. The different components can be either on the molecular or micro scale such as segments of block copolymers or components of macromolecules. A diagram depicting the differences between the two mechanisms is seen in Figure 1. Finally, the third mechanism is the partial transition, where a mixture of two materials—one of which changes phases—controls the shape memory effect where the example given by Yang et al. [26] was a compressible sponge infiltrated by paraffin wax, which transformed from a solid to liquid upon the application of heat.

The observation of phase alignment and the effect of phase alignment on shape memory properties in our previous work presented in Chávez et al. [22] also set a premise that we used as a template for the design of SMP materials for extrusion-based AM platforms. We feel that, in order to take full advantage of the FFF process, immiscible blends should be used in order to facilitate the presence of phases that can be aligned during the component manufacturing process. Miscibility of polymer mixtures can be estimated in part by the Hildenbrand solubility parameter (δ) and taking into account the constituents used in our previous work, ABS has a solubility parameter ranging between 20 and 23 MPa^1/2^ [28] whereas SEBS has a solubility parameter of ~17 MPa^1/2^ [29] and would therefore result in an immiscible blend when combined. Further characterization via scanning transmission electron microscopy (STEM) confirmed the immiscibility of these polymer constituents. 

The work presented here is part of a larger body of work found in reference [27]. An initial report of this research effort was presented by Quiñonez et al. [6], where it was pointed out that the solubility parameters (δ) are similar for PLA and TPU at values of 20 to 20.5 MPa^1/2^ [30,31]. Recalling again that the solubility parameter of SEBS is ~17 MPa^1/2^ [29], the blending of PLA and SEBS would be expected to result in an immiscible blend while PLA and TPU would be expected to be more compatible. For the effort presented here, we purposely chose to combine materials with different shape memory mechanisms. Our intent was twofold as we desired to: (1) attempt to determine which shape memory mechanism was more dominant; and (2) attempt to create a material system with three-way shape memory capabilities—an ability to transform between more than two shapes during a shape memory cycle. PLA is a relatively common 3D printable material particularly among desktop grade FFF systems. On its own, PLA has shape memory properties driven by the dual state mechanism. As mentioned above, TPU also has shape memory properties, however the mechanism of shape memory effect is dual component. The co-polymer triblock SEBS has been shown to exhibit shape memory properties [32] that are also driven by a dual component mechanism due to the hard and soft block copolymer components. Further, SEBS has been used to create shape memory polymeric material systems by others [33,34] in addition to the work previously carried out by our group [22]. Based on the results of previous works conducted by our group and others we created two distinct systems of materials, PLA:SEBS and PLA:TPU. Efforts exploring the shape memory properties of similar material systems can be found in literature. Combining a polyamide elastomer (PAE) to PLA was demonstrated by Zhang et al. [35] to enhance the inherent shape memory properties of the biopolymer. Modification of the toughness and heat resistance of PLA by the addition of SEBS in the creation of PLA:SEBS blends has also been demonstrated in literature, but the shape memory performance of this combination of thermoplastics was not explored [36,37]. Our work also differs from prior works found in literature as those efforts did not involve the use of AM. 

In the work presented here, we seek to not only determine which shape memory mechanism is more dominant, but also further explore the effect of phase morphology and alignment on shape memory performance. By comparing additively manufactured test specimens with injection molded specimens, we seek to decouple the effect of raster pattern on mechanical properties, which has been documented heavily in literature [2,38,39,40,41], from that of phase morphology. The work presented here also demonstrates a process of developing novel thermoplastic material systems for AM processing, in this case, technologies based on the FDM platform.

## 2. Materials and Methods 

Both of the blend systems characterized in this study were based on PLA, supplied by NatureWorks, LLC (Ingeo Biopolymer Grade 4043D, NatureWorks, LLC, Minnetonka MN, USA). Grade 4043D was chosen as this particular grade is considered to be a pure form of PLA and does not contain additives such as crystallization promoters or impact modifiers [42]. Thermoplastic elastomers were added to PLA in increasing weight percentages (5%, 10%, 25%, and 50%). As was the case in the shape memory studies used in previous efforts by our group, maleated SEBS (SEBS-g-MA) supplied by Kraton (grade FG1901-GT, Kraton, Houston, TX, USA) was combined with PLA in the creation of one PLA/elastomer blend. The other blend system consisted of combining TPU with PLA. The TPU used was NinjaFlex (Fenner, Inc. Manheim, PA, USA) which was acquired in the form of a 1.75 mm diameter 3D printer filament and classified as natural by the manufacturer, meaning that no dyes had been added to the material. To facilitate blending, the filament was first pelletized by a Collin Teachline strand pelletizer (Collin Lab and Pilot Solutions, Norcross, GA, USA). Both blend systems were compounded in a Collin twin screw extruder (Model ZK-25T) with co-rotating, intermeshing screws. Filaments of each material composition were extruded to a 2.85 mm target diameter in order to be compatible with our FFF-type 3D printers. Prior to extrusion, the pellets were dried in a compressed air dryer (Dri-Air CFAM Micro-Dryer, East Windsor, CT, USA).

**Figure 2:**
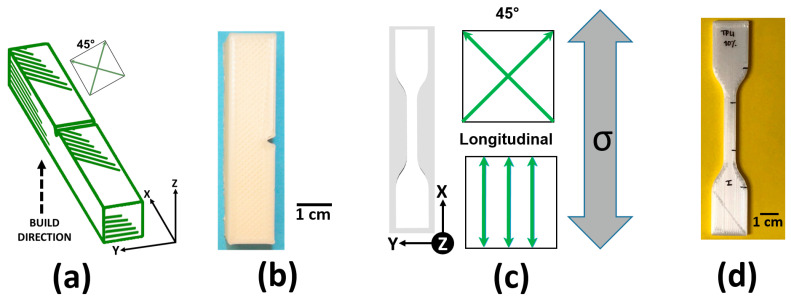
The mechanical testing specimen types used in this study: (**a**) schematic of an Izod impact test specimen with print orientation and raster pattern details indicated, (**b**) example of an additively manufactured Izod impact test specimen used in this study, (**c**) schematic of an ASTM D638 [44] Type IV tensile test specimen with print orientation and raster pattern details indicated as well as relation to applied stress, and (**d**) example of an additively manufactured Type IV tensile test specimen used in this study.

Three test specimen types were additively manufactured with a Lulzbot Taz series 3D printer (Aleph Objects, Loveland, CO, USA): tensile test specimens following the ASTM D638 [43] Type IV specimen geometry, Izod impact test specimens following the geometrical specifications indicated in the ASTM D256 standard [44], and specimens for dynamic mechanical analysis (DMA) according to the ASTM D4065 standard [45]. For tensile test specimens, two raster patterns were explored, an alternating 45° crosshatched raster pattern, and a longitudinal raster pattern where all print rasters were parallel to the direction of applied stress. Only the 45° raster pattern was used for the impact test specimens as this raster pattern has been found by our group to exhibit the best resistance to impact for a variety of thermoplastics [46]. A depiction of the raster pattern scheme for the test specimens used in this study are seen in Figure 2. All specimens were printed with a layer height of 0.2 mm and an infill percentage of 100%. Additional specimens for tensile and impact testing were created via injection molding on an LNS Technologies manual injection molder (Model 150A, LNS Technologies, Scotts Valley, CA, USA). Processing parameters for the specimen fabrication via AM and injection molding of each material type are seen in Table 1 and Table 2, respectively. In the case of the print parameters presented in Table 1, it can be seen that increasing elastomer content necessitated an increase in print temperature due to an increase in viscosity.

Tensile testing as well as deformation of specimens for shape memory characterization was carried out on an MTS Criterion C-44 tensile testing machine outfitted with an Advantage™ Model AXH800 extensometer (MTS Systems Corporation, Eden Prairie, MN, USA). Impact testing was performed through the use of a Tinius Olsen IT-504 polymer impact tester (Tinius Olsen, Horsham, PA, USA) with a pendulum weight of nominal weight of 1553.5 +/− 7.6 g and latched pendulum potential energy of 7.44 J. A PerkinElmer Model DMA 8000 (PerkinElmer, Waltham, MA, USA) was used to determine the onset of glassy behavior as well as maximum loss tangent (tan δ). The DMA testing was performed over a temperature range of −40 to 110 °C at a rate of 5 °C/min and a frequency of 1 Hz. The presence of crystalline domains within the blend systems was confirmed by analyzing X-ray diffraction spectra generated by a Bruker D8 Discover X-Ray Diffractometer (Bruker Scientific LLC, Billerica, MA, USA) equipped with a Cu K-α (λ = 1.54 Å) source. Electron microanalysis was performed through the use of a Hitachi SU-3500 (Hitachi America, Ltd., New York, NY, USA) scanning electron microscope (SEM) outfitted with a backscatter electron (BSE) detector as well as an ultra-variable detector (UVD). Mitigation of electron charging effects was achieved by operating the SEM in variable pressure mode with a vacuum of 90 Pa. The phase morphology of certain blends was characterized on the same SEM system by using an auxiliary STEM unit (Deben UK Ltd., Suffolk, UK). Thin sections were prepared by cryo-ultramicrotomy using an RMC PT-X ultramicrotome (Boeckeler Instruments, Tucson, AZ, USA) with a CR-X cryo-sectioning unit and RMC wet cryo diamond knife. 

The experimental methodology for the characterization of shape memory properties was to first perform tensile testing on all material types whether they were manufactured by AM or injection molding (IM). Those materials that demonstrated an ability to withstand strain of greater than 100% elongation were selected for shape memory characterization. We lacked the ability to deform the materials at an elevated temperature so room temperature deformation was used. The tensile test specimens were then recovered in a forced air oven (Model 3.65, VWR International, Radnor, PA, USA). The recovery temperature was determined based on the max tan δ obtained from DMA testing as will be explained further below. 

## 3. Results

### 3.1. Initial Characterization of the Blends

**Figure 3:**
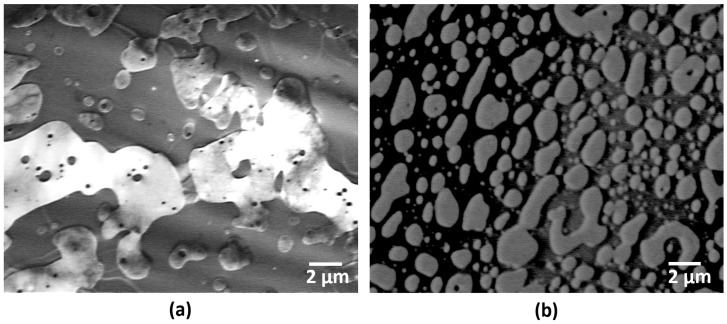
STEM micrographs of (**a**) PLA:SEBS and (**b**) PLA:TPU where both blends are of a 50:50 by weight composition.

Scanning transmission electron micrographs of the 50:50 compositions revealed aspects related to the mixing behavior of the polymer constituents. As can be seen in Figure 3, both combinations exhibited characteristics of immiscible blends. Two distinct phases are visible in the micrograph of the PLA:SEBS mixture (Figure 3a) where the two phases appear to be semicontinuous. The blend composed of PLA:TPU exhibited a differing morphology where the TPU is more uniformly dispersed within the PLA. We based our phase identification on the morphology of a similar PLA:TPU blend characterized by Lai and Lan [23]. Based on this identification, we are also asserting that the lighter contrast phase observed in the PLA:SEBS blend is SEBS as we believe that PLA would exhibit the same contrast mechanisms when observing either blend. Though the phase distribution was not uniform for the PLA:SEBS blend, it is believed that the presence of a maleic anhydride graft would promote compatibility between the two constituents. 

**Figure 4:**
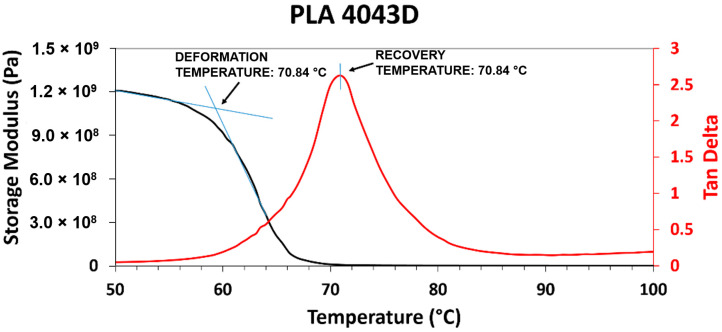
Example of determination of the thermal schedule for shape memory property characterization, in this case, for PLA.

Dynamic mechanical analysis of the two blend systems was carried out to determine the recovery temperature for shape memory characterization. When developing temperature schedules for shape memory polymers, we have found it best to perform high temperature deformation near the glassy onset (as determined by storage modulus drop-off from DMA curves) and the recovery temperature to be determined by the max tan δ temperature [6,13,22]. An example of this process is shown in Figure 4 for PLA Grade 4043D. Since, for this study, we were unable to perform elevated temperature deformation, the pertinent information related to shape memory property assessment from the DMA curves was the max tan δ temperature. For 5% SEBS, 10% SEBS, 25% SEBS, and 50% SEBS, we obtained a tan δ of approximately 1.65, 1.54, 1.14, and 0.64, respectively. For PLA combined with by weight percentages of 5% TPU, 10% TPU, 25% TPU, and 50% TPU, we obtained maximum tan δ values of 1.51, 1.59, 1.35, and 0.55, respectively. In both cases, the addition of an elastomeric material to PLA led to an overall increase in dampening ability. There was a slight increase in tan δ for 10% TPU in comparison to other blends. It was also noted that the temperature at which the maximum tan δ was reached was increased as compared to neat PLA (71 °C) and a range of 77 to 81 °C. Max tan δ values and the temperatures at which they were observed for all blends evaluated in this study are listed in Table 3.

**Figure 5:**
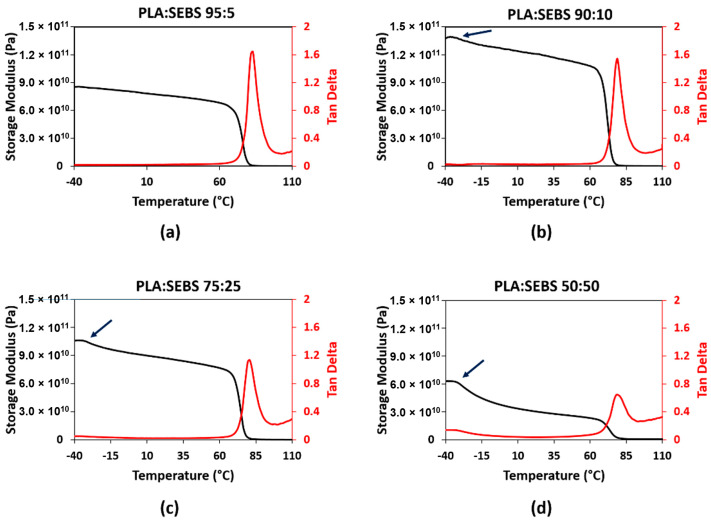
DMA curves for the PLA:SEBS blend system where (**a**–**d**) correspond to 5, 10, 25, and 50% by weight SEBS, respectively.

**Figure 6:**
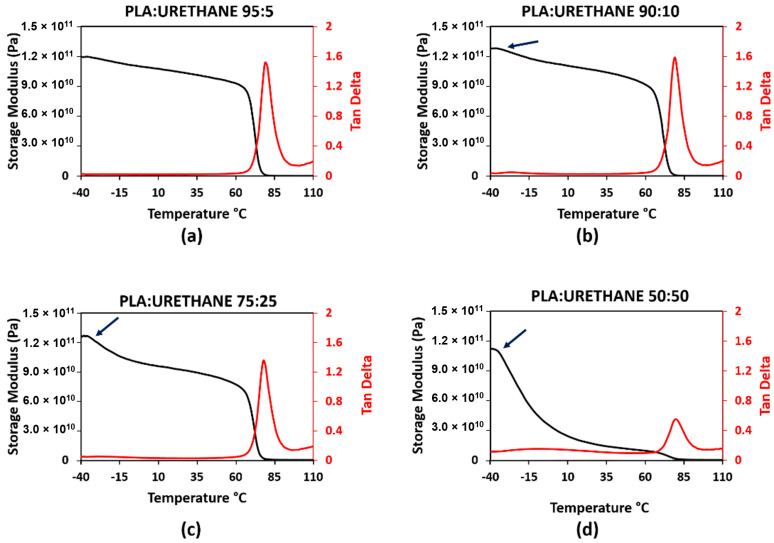
DMA curves for the PLA:TPU blend system where (**a**–**d**) correspond to 5, 10, 25, and 50% by weight TPU, respectively.

The glass transition temperature of a miscible binary polymer blend has been demonstrated elsewhere to be predicted by Fox’s law, which is defined by the equation:

(4) $$\frac{1}{T_{g\;Blend}}=\frac{x_{1}}{T_{g1}}+\frac{x_{2}}{T_{g2}}$$

where *T_g_*_1_ and *T_g_*_2_ and *x*_1_ and *x_2_* are the glass transition temperatures and weight fractions of the individual constituents, respectively [47]. However, evaluation of the temperatures at which the max tan δ occurs reveals the glass transition behavior of the blend systems studied here not to follow Fox’s law—further enforcing the fact that neither combination of polymers results in a miscible blend. Values of Fox’s law calculations considering the published *T_g_* values of −10, −40, and 60 °C for the TPU, SEBS, and PLA grades used here are tabulated in Table 3. Moreover, further scrutinization of the DMA curves (Figure 5 and Figure 6) reveals two glassy onset temperatures that become more prominent with an increase in elastomer content for both blend systems, effectively indicating the presence of two glass transition temperatures, which are tabulated in Table 3. 

**Figure 7:**
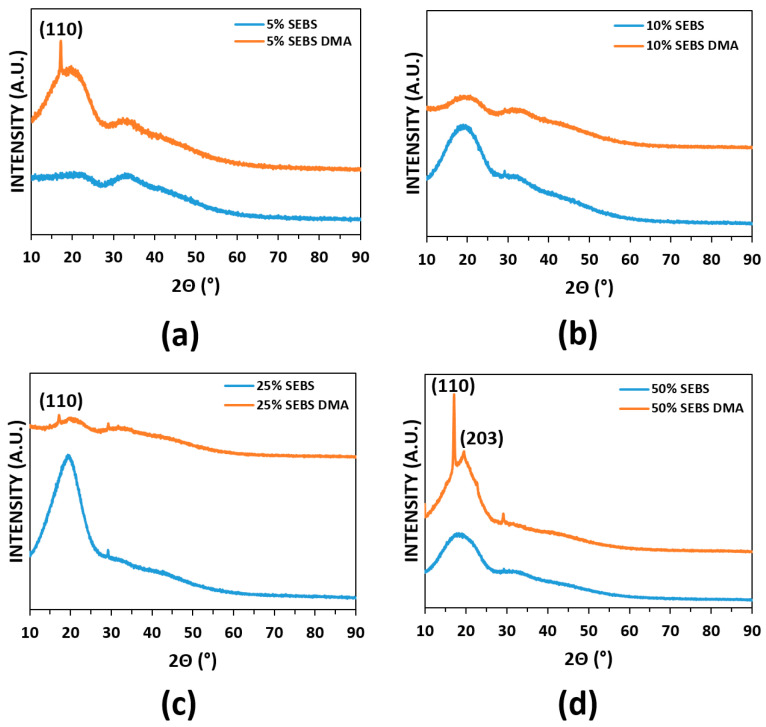
XRD spectra of the PLA:SEBS blend combination before and after DMA testing where (**a**–**d**) correspond to 5, 10, 25, and 50% by weight SEBS, respectively.

**Figure 8:**
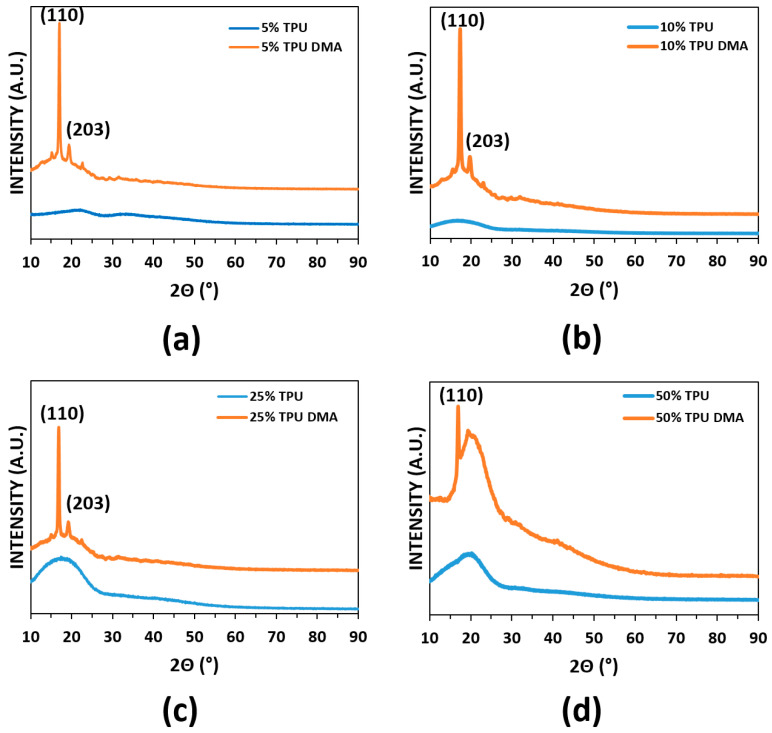
XRD spectra of the PLA:TPU blend combination before and after DMA testing where (**a**–**d**) correspond to 5, 10, 25, and 50% by weight TPU, respectively.

Prior to DMA testing, specimens were examined via XRD to ascertain whether the blends exhibited crystallinity. Initial scans revealed that all blend compositions were amorphous in the as-printed condition. We sought to determine the effect of thermomechanical cycling on crystallinity so we repeated the XRD analysis after performing DMA testing. We found that the DMA test imparted a level of crystallinity for every blend combination with the exception of the PLA:SEBS 90:10 by weight combination. It is known that PLA can be annealed to manifest crystallinity with a known (110) peak present at roughly 16.6° and the (203) peak visible at roughly 19.0° [42]. The (110) reflection is visible on most of the XRD spectra seen in Figure 7 and Figure 8. The effect of DMA on the manifestation of crystalline peaks was more prominent for the PLA:TPU blend system to the point that additional reflections, namely the (203) peak, became prominent. It is not fully understood why the PLA:SEBS 90:10 combination did not manifest crystalline domains due to DMA cycling, nor is it understood why the prominence of crystalline peaks was more prominent for the TPU blends. The presence of crystalline peaks was not expected, as a common annealing schedule for PLA is 120 °C for twenty minutes [42,48]; below the maximum temperature of our DMA testing. The presence of crystallinity may also be strain induced in the work presented here.

### 3.2. Mechanical Testing

**Figure 9:**
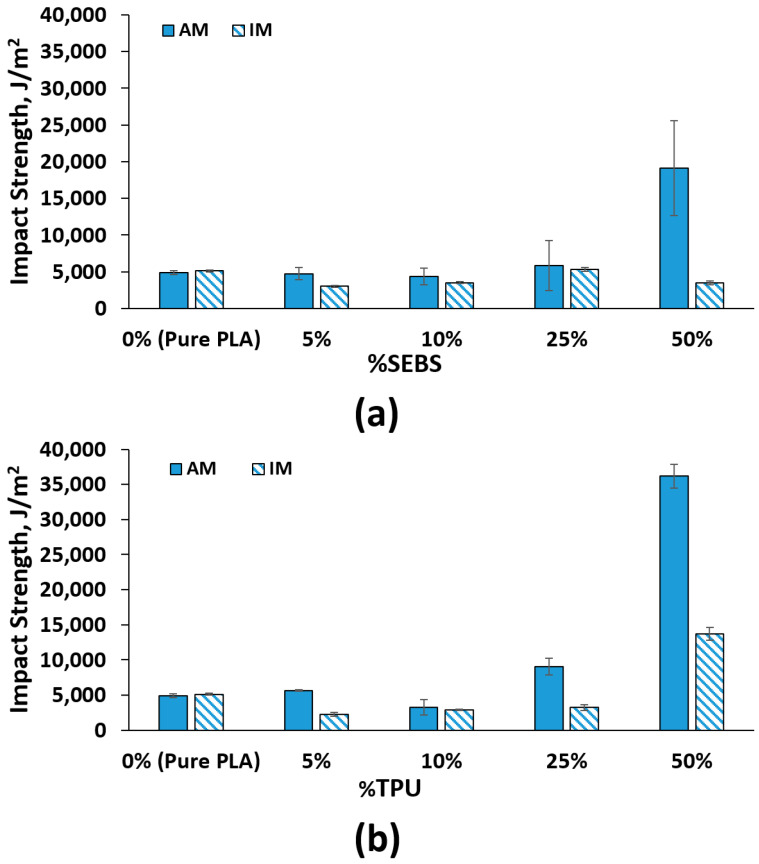
Impact strength values for (**a**) the PLA:TPU system and (**b**) the PLA:SEBS system where AM corresponds to additively manufactured specimens and IM corresponds to injection molded specimens.

The effect of the addition of elastomeric materials to PLA on the impact strength of additively manufactured Izod test specimens is evident, particularly at the 25% and 50% by weight ratio blends for both systems. Injection molding of Izod impact test specimens proved to be difficult due to the manifestation of voids in the center of the specimen. The voids occurred due to the style of injection molding machine being a plunger-based system rather than a screw based system. This, in combination with the thickness of the Izod test specimen (12 mm × 12 mm), made a viable comparison of impact strength between the two manufacturing methods impossible. Screw-based injection molding systems provide better distribution of heat within the polymer pellets, negating voids. Nevertheless, we have included the injection molded data in the graphical results in Figure 9. Overall, the addition of TPU had a greater effect on improving the impact strength for the 25 and 50% blend compositions. The reason behind the greater efficacy of TPU acting as an impact modifier for PLA as compared to SEBS may be due to the more uniform distribution of the TPU phase within PLA.

**Figure 10:**
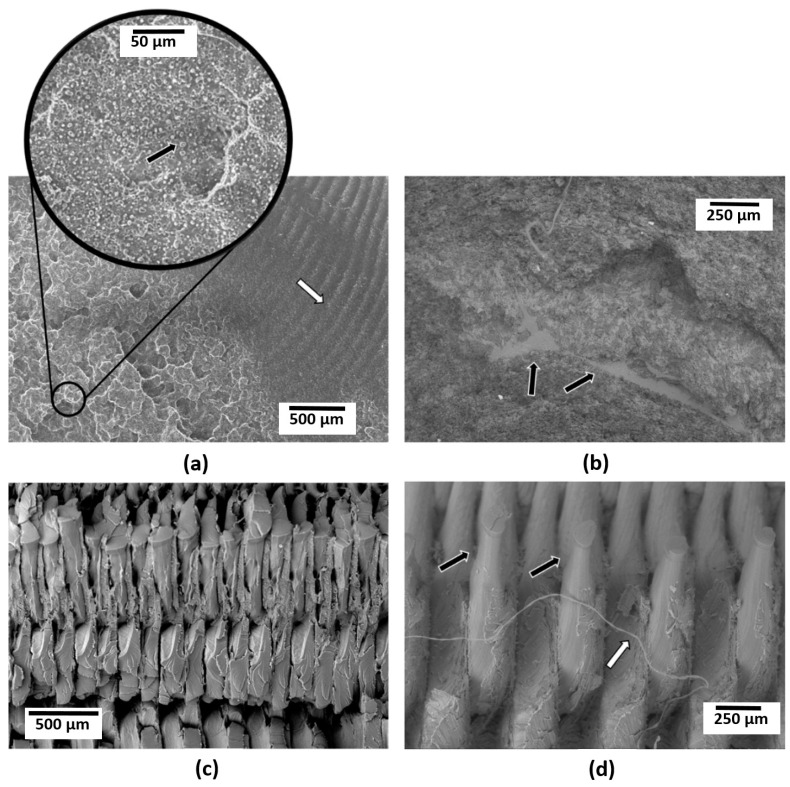
Scanning electron micrographs of the impact test fracture surfaces of (**a**) an injection molded specimen composed of PLA:TPU 50:50, (**b**) an injection molded specimen composed of PLA:SEBS 50:50, (**c**) a FFF-manufactured specimen composed of PLA:TPU 50:50, and (**d**) a FFF-manufactured specimen composed of PLA:SEBS 50:50. Note the differences in fracture surface morphology.

The injection molded specimens did, however, provide additional information pertinent to this study. Scanning electron microanalysis of injection molded specimens revealed aspects related to the mixing of each individual blend system. Comparing the 50:50 compositions of each blend systems (Figure 10) indicates that the TPU formed a well-dispersed phase within a PLA matrix (indicated by the black arrow in the inset in Figure 10a) whereas the SEBS-g-MA formed a continuous phase along with the PLA. The PLA is discernible in Figure 10b due to the smother, brittle mode fracture surface morphology. The PLA:TPU specimen also exhibited different types of ductile fracture as areas of a high amount of plastic deformation were adjacent to striations (indicated by the white arrow in Figure 10a). In contrast, the FFF process obscured differences in phase morphology. The electron micrographs of PLA:TPU and PLA:SEBS (Figure 10c,d, respectively) indicate differences in the material response to impact, as the PLA:SEBS blend exhibited necking of the individual print rasters (indicated by the black arrows in Figure 10d) as well as a fibril (designated by the white arrow in Figure 10d) indicating that the PLA:SEBS blend was more ductile than the PLA:TPU blend. 

**Figure 11:**
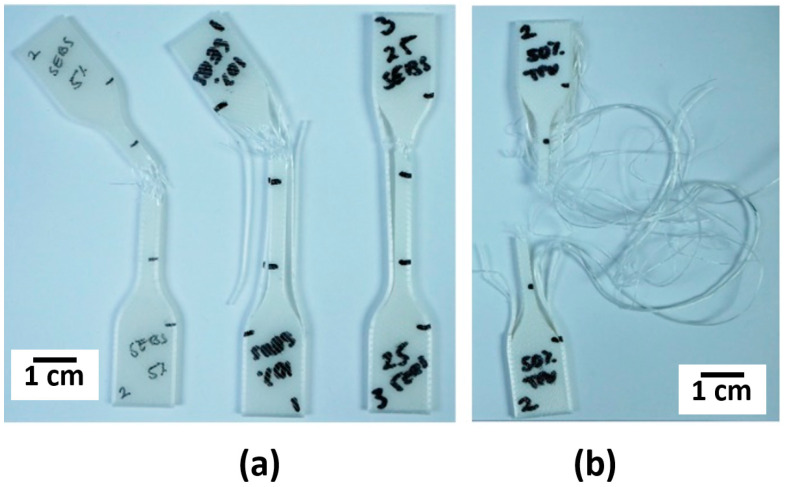
Examples of delamination of a FFF specimens fabricated in a 45° raster patterns in the case of (**a**) PLA:SEBS blends and (**b**) PLA:TPU.

Tensile testing was performed to not only ascertain the stress-strain performance, but to act as a stopgap to determine which blend systems would be able to sustain 100% elongation at room temperature. Achieving 100% elongation prior to rupture was found to not be the only metric of importance as the key inhibitor to a specimen becoming a successful candidate for shape memory evaluation ended up being delamination of FFF-fabricated components as seen in Figure 11. The 45° raster pattern was prone to delamination for all experiment sets making this raster pattern unsuitable for room temperature shape memory experiments for both material systems. Additionally, none of the FFF-fabricated PLA:SEBS specimens were found to be suitable shape memory experimentation. For injection molded experiments involving the PLA:SEBS blend system, only the 50% composition was found to be able to sustain 100% elongation at room temperature. 

**Figure 12:**
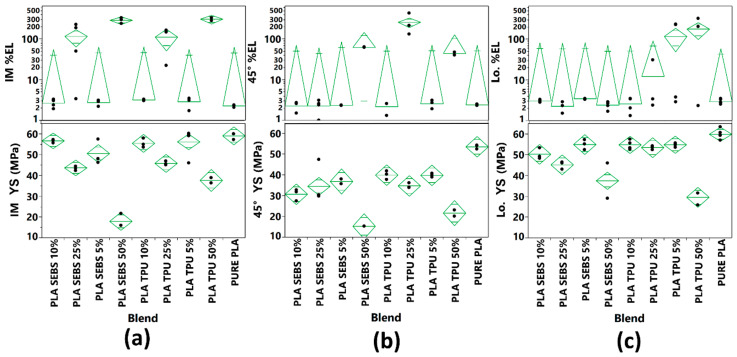
Tensile test data for the blend systems evaluated here for (**a**) injection molding (IM), (**b**) FFF in a 45° raster pattern, and (**c**) FFF in a longitudinal (Lo.) raster pattern.

In the case of PLA:TPU fabricated by FFF, only the 5, 25, and 50% passed the 100% elongation stopgap. We included the 10% by weight TPU experiment set because though the machine did not record 100% elongation, the specimen was somewhat intact at this elongation value. In the case of injection molded specimens, only the 50% by weight TPU experiment set was able to reliably sustain 100% elongation. It is noteworthy that, in the case of PLA:TPU, more experiment sets were able to sustain 100% elongation as compared to those fabricated via injection molding. Tensile test data including yield strength (YS) and % elongation at break is graphically represented in Figure 12. 

### 3.3. Shape Memory Characterization

**Figure 13:**
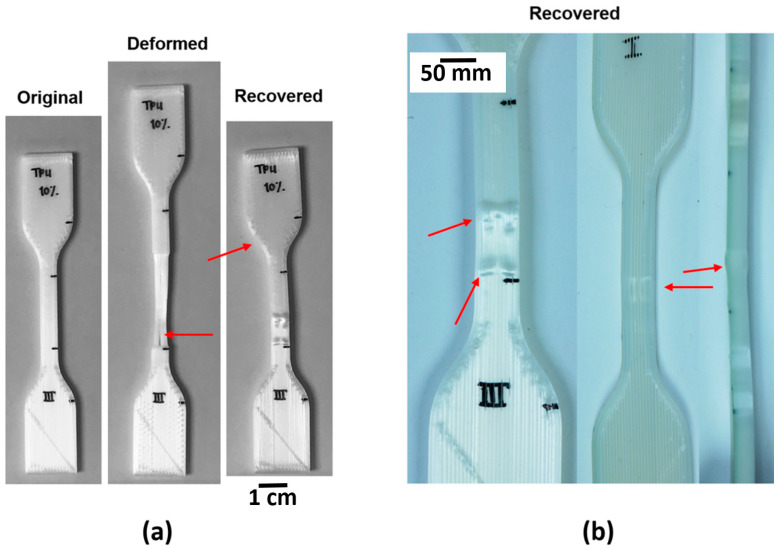
Examples of damage and distortion that occurred (**a**) during the deformation and recovery process. (**b**) Higher magnification image depicting the damage that was observed after the recovery process. In this case, the images are of PLA:TPU FFF-manufactured in a 90° raster pattern.

**Figure 14:**
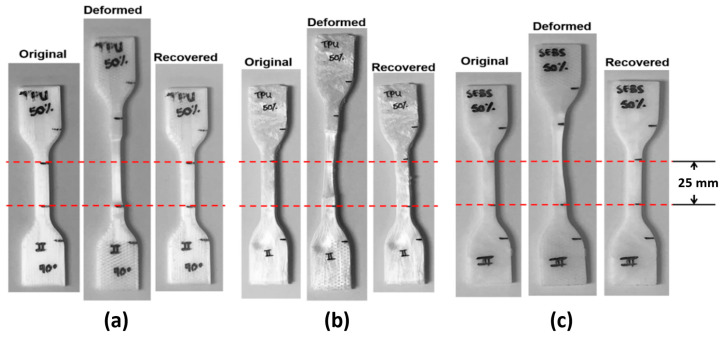
Examples of the process used to determine shape memory properties for (**a**) PLA:TPU 50:50 FFF-manufactured in a longitudinal raster pattern, (**b**) PLA:TPU 50:50 injection molded, and (**c**) PLA:SEBS 50:50 injection molded.

Systematic characterization of the shape memory properties was carried out on the blends and manufacturing methods that passed the stopgap test. A surprising result was that all of the material sets subjected to shape memory characterization exhibited fixation ratios greater than 90% and recovery ratios of ~100%. Caveats that need to be mentioned related to the measurements that led to the calculations of the ratios include: (1) measurements were manually made with a micrometer from marks made in the gage section of the specimen; (2) the marks were, themselves, manually placed with a Pilot Super Color marker (Pilot Corporation of America, Trimbull, CT, USA); (3) some of the samples were damaged or distorted during the deformation and recovery processes (see red arrows in Figure 13), respectively; and (4) the sample pools the measurements were taken from are small (*n* = 2). An example of the measurement process is seen in Figure 14. The characteristic shape memory values of *R_f_*, *R_r_*, and *SMI* are tabulated in Table 4. In terms of SMI, the best performing material/manufacturing method was the PLA:TPU blend in a 50:50, by weight ratio fabricated by FFF in a longitudinal raster pattern.

**Figure 15:**
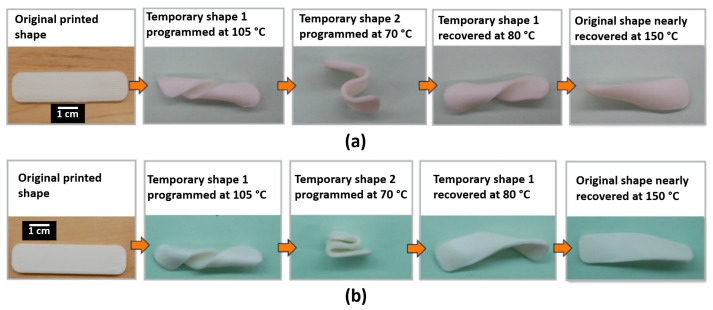
Manual shape memory experiments with the goal of achieving multiple programmed temporary shapes for (**a**) PLA:SEBS 50:50 and (**b**) PLA:TPU 50:50. The specimens were FFF-manufactured.

Realizing the temperature control-related limitations of our tensile testing apparatus, we devised an elevated temperature experiment using a non-standard specimen that was manufactured by FFF in a longitudinal raster pattern (Figure 15) using the PLA:SEBS and PLA:TPU blends, both in a 50:50, by weight ratio. For this experiment, we sought to understand a research question; since our material systems were essentially two-phase mixtures, each with inherent shape memory characteristics of their own, could multiple shapes be programmed into a specimen? We first experimented with PLA:SEBS 50:50. The original printed shape would act as the parent shape as it was printed at 250 °C for PLA:TPU and 260 °C for PLA:SEBS. We chose our first deformation temperature to be 105 °C as that was the deformation temperature used for high temperature deformation in our previous work by Chávez et al. [22]. The specimen was heated in an oven to 105 °C, deformed manually into a twist shape, and then allowed to cool to room temperature. The specimen was then reheated to 70 °C, a temperature chosen as it was below the max tan δ of the blends and just below the max tan δ value of pure PLA Grade 4043D. The specimen was manually manipulated to an “S” shape at 70 °C and then allowed to cool to room temperature. The specimen was then recovered in an oven at 80 °C and the specimen returned to the “S” shape, though not a total recovery. The specimen was then allowed to cool back down to room temperature. Finally, in an effort to recover the original shape, the specimen was heated to 150 °C, resulting in nearly the original shape. The process was repeated for PLA:TPU, where recovering the temporary shape programmed at 105 °C was not as effective, potentially indicating that a different temperature schedule was necessary. The sequence of events were documented and are presented in Figure 15a,b for PLA:SEBS and PLA:TPU, respectively. 

## 4. Discussion

A key aspect of this work was that, for the PLA:TPU blend system, the FFF AM process had a positive effect on the mechanical properties of the material. Though the 45° raster pattern was not able to be used for SMP characterization, the longitudinal raster pattern was able to sustain 100% elongation at room temperature for most of the experiment sets evaluated here. A true head-to-head comparison was feasible for the PLA:TPU 50:50 blend combination between injection molding and the FFF process. In terms of SMI, the FFF-made specimens had a greater value of 99.34 ± 0.62% compared to 96.37 ± 1.57% for IM specimens. Our previous work with two component SMP material systems involving ABS:SEBS blends also found the longitudinal raster pattern to be superior when compared to other FFF raster patterns and the alignment of phases by the FFF process was believed to be the mechanism by which this occurred. However, other aspects of the FFF process such as interlayer adhesion between print beads acted as evidence against this notion. By making a comparison between FFF and injection molding, the work presented here serves to deconvolute other potential variables related to the FFF process and further supports the idea that texturized microstructure plays a key role in SMP performance. 

In terms of design of shape memory materials for FFF platforms, developing two-component systems is an advantageous strategy due to the texturization of phase domains imparted by the manufacturing process. Utilizing the miscibility parameter as a guide in the design of multi-component polymer systems is also beneficial as demonstrated here by the differences observed between PLA:SEBS and PLA:TPU systems where PLA and TPU had more closely matched miscibility parameters. Though we were limited in terms of our ability to systematically deform specimens at temperatures greater than room temperature, the work presented here provides insights into designing thermoplastic shape memory polymeric systems for the additive manufacturing process of fused filament fabrication. Further experimentation is needed in order to determine which shape memory mechanism is more dominant. 

## 5. Conclusions

Designing materials for additive manufacturing platforms should be done in a way that takes advantage of physical attributes imparted to the material by the process itself. In the example presented here, the FFF process is known to align the individual phases in multi-component polymeric systems, leading to an improvement in shape memory properties. Thus, novel shape memory polymer systems for FFF platforms should be designed to be multi-phase. Another advantage of utilizing multi-component polymer systems is the potential to develop material systems that can hold multiple temporary shapes that can be imparted at multiple switching temperatures.

The materials systems presented here present potential usefulness in the area of so-called “4D Printing” and could potentially benefit from an in-depth optimization of machine parameters for various FFF platforms. Additionally, as alluded to above, refinement of temperature schedules for multi-shape transformation is warranted to allow these systems to be utilized in applications that could benefit from more than “two-way” shape memory processes. Other avenues of exploration that have opened up include the potential strain-induced crystallinity of the material systems evaluated here. Designing material systems such as shape memory polymers for FDM™-type AM processes serves to further the applicability of this manufacturing method as well as open the door to novel material discoveries. 

## Figures and Tables

**Table 1:** Printer parameters for the blends used in this study ^1^.

Material System	PLA:TPU				PLA:SEBS			
**Ratio**	5:95	10:90	25:75	50:50	5:95	10:90	25:75	50:50
**Nozzle Temp. (°C)**	205	212	225	250	205	210	225	260
**Bed Temp. (°C)**	60	60	60	60	60	60	60	60
**Printing Speed (mm/s)**	30	30	30	30	30	30	30	30

^1^ Pure PLA specimens were fabricated with the same parameters as the 5:95 blend ratios.

**Table 2:** Injection molding temperatures for the blends used in this study ^1^.

Material System	PLA:TPU				PLA:SEBS			
**Ratio**	5:95	10:90	25:75	50:50	5:95	10:90	25:75	50:50
**Injection Temp. (°C)**	190	190	200	200	190	190	200	200

^1^ Pure PLA specimens were fabricated with the same parameters as the 5:95 blend ratios.

**Table 3:** Glassy onset temperatures and maximum loss tangent values.

Blend	Glassy Onset Temp. 1 (°C)	Glassy Onset Temp. 2 (°C)	Max tan δ	Max tan δ Temp. (°C)	Calculated *T_g_* (°C)
PURE PLA	N/A	60	2.63	71	--
SEBS 5%	N/A	70	1.65	81	53.00
SEBS 10%	−35	70	1.54	80	46.30
SEBS 25%	−34	70	1.14	80	27.73
SEBS 50%	−33	68	0.64	78	1.17
TPU 5%	N/A	67	1.51	77	55.63
TPU 10%	−35	66	1.59	77	51.37
TPU 25%	−34	66	1.35	77	39.22
TPU 50%	−37	68	0.55	77	20.89

**Table 4:** Shape memory property values for the material systems that were subjected to a deformation/recovery cycle.

Blend	Manufacturing Method	*R_f_* (%)	σ	*R_r_* (%)	σ	SMI (%)	σ	Sample Size (*n*)
SEBS 50%	IM	97.17	2.35	100.00	0.00	97.17	2.35	2
TPU 50%	IM	96.37	1.57	99.9	0.00	96.37	1.57	2
TPU 50%	FFF Long.	99.34	0.00	99.9	0.00	99.34	0.62	2
TPU 25%	FFF Long.	96.95	2.33	100.00	0.00	96.95	0.00	2
TPU 10%	FFF Long.	98.52	0.64	99.9	0.00	98.52	0.00	2
TPU 5%	FFF Long.	97.48	1.82	100.00	0.00	97.48	1.82	2

## Data Availability

Data sharing is not applicable to this article.
